# Medical Graduations at Edinburgh, 1836

**Published:** 1837-01

**Authors:** 


### MEDICAL GRADUATIONS AT EDINBURGH, 1836.

On the 1st of August, 1836, the Senatus Academicus of the University of Edinburgh conferred the degree of Doctor of Medicine on 126 candidates, after having gone through the appointed examinations, and publicly defended their inaugural dissertations. The list contained fifty gentlemen from Scotland; twenty-seven from England; three from Wales; twenty-seven from Ireland; and sixteen from our colonies and foreign countries. All the dissertations were in English, except one, which was in French. Our old associations are shocked by the absence of *Latin* in these exercitations; and we cannot persuade ourselves that the regulations which exclude this classical language—or, which seems to be the same thing, do not render its use imperative—are not calculated to lead to a deplorable decrease of classical knowledge. Admitting the propriety of the chief examinations being in English, surely there was no necessity for banishing Latin from the thesis.

